# Benefits of pulmonary rehabilitation in patients with advanced lymphangioleiomyomatosis (LAM) compared with COPD – a retrospective analysis

**DOI:** 10.1186/s13023-020-01540-3

**Published:** 2020-09-22

**Authors:** Rainer Gloeckl, Christoph Nell, Tessa Schneeberger, Inga Jarosch, Martina Boensch, Henrik Watz, Hubert Wirtz, Tobias Welte, Klaus Kenn, Andreas Rembert Koczulla

**Affiliations:** 1 Department of Pulmonary Rehabilitation, Philipps - University of Marburg, German Center for Lung Research (DZL), Marburg, Germany; 2 Institute for Pulmonary Rehabilitation Research, Schoen Klinik Berchtesgadener Land, Malterhoeh 1, 83471 Schoenau am Koenigssee, Germany; 3 Department of Internal Medicine, Division of Pulmonary Diseases, Philipps University of Marburg, Marburg, Germany; 4 Pulmonary Research Institute at Lungen Clinic Grosshansdorf, Airway Research Center North (ARCN), Member of the German Center for Lung Research (DZL), Grosshansdorf, Germany; 5 Department of Respiratory Medicine, University of Leipzig AöR, Leipzig, Germany; 6 Department of Respiratory Medicine, Hannover Medical School, Hannover, Germany; 7 Teaching Hospital, Paracelsus Medical University, Salzburg, Austria

**Keywords:** Lymphangioleiomyomatosis, LAM, Pulmonary rehabilitation, Exercise, Quality of life, Therapy

## Abstract

**Abstract:**

Lymphangioleiomyomatosis (LAM) is a rare and progressive cystic lung disease with limited therapeutic options. We retrospectively analyzed the effects of a comprehensive 4-week inpatient pulmonary rehabilitation (PR) program in 58 patients with advanced LAM (FEV1: 45 ± 34%predicted, 6-min walk distance (6MWD): 338 ± 167 m). Exercise performance (6MWD: + 49 ± 50 m; *p* < 0.001) and quality of life (SF-36 physical component: + 2.4 ± 7.8 points; *p* = 0.049 and mental component: + 5.2 ± 12.1 points; *p* < 0.001) increased significantly after PR comparable to an COPD cohort. There were no clinical parameters that predicted changes in outcomes following PR. PR seems to be an effective therapeutic option even in patients with advanced LAM.

**Trial registration:**

Clinical-Trials registration number: NCT04184193; date of registration: December 3, 2019.

## Introduction

Lymphangioleiomyomatosis (LAM) is a very rare, systemic neoplastic disease associated with progressive cystic lung destruction mostly affecting young women (prevalence: 3.4 to 7.8 per million women) [1]. LAM results in airflow limitation, hyperinflation, and reduced diffusion capacity which in turn leads to dyspnea and impaired exercise performance, physical activity, and quality of life [2, 3]. Available drugs (mTOR-inhibitor) may slow down lung destruction, however, the disease remains incurable and lung transplantation is often the only therapeutic option [1, 4]. Similar to other chronic lung diseases pulmonary rehabilitation (PR) might improve LAM patients´ symptoms and limitations [5]. Since LAM is a rare disease data collection is difficult and therefore, we performed a retrospective analysis of LAM patients that were referred to PR.

## Methods

Data for this analysis were consecutively collected between July 2000 and November 2019 during a 4-week inpatient PR program at the Schoen Klinik Berchtesgadener Land (Schoenau, Germany). Patients performed a comprehensive multimodal, multidisciplinary PR program with contents specialized for patients with chronic respiratory diseases. The program was provided on 5 to 6 days per week and consisted of daily exercise training sessions (including endurance and strength training for 60 min) following recommendations for exercise training in COPD [6]. Patients participated also in structured general education sessions (e.g. disease management or oxygen therapy) and respiratory physiotherapy – smoking cessation, nutritional and psychological counseling were provided on a case by case basis.

This retrospective analysis was approved by the Ethics Committee of the Philipps-University Marburg (ID EK_MR_26_11_2019_koczulla) and is registered at ClinicalTrials.gov (NCT04184193).

### Statistical analyses

The primary outcomes were changes in 6-min walk distance (6MWD) [7] and quality of life (short-form 36 question health survey [SF-36]). Results were provided by mean values +/− standard deviation. For comparing pre to post PR, a two-tailed Wilcoxonrank sum test (W-test) was applied. The Mann-Whitney U-test(U-test) was used to compare the two groups at the beginning and the delta values (pre to post PR) with a significance level of *p* < 0.05. A logistic regression analysis was performed to identify the relationship between various baseline characteristics on changes in 6MWD and the physical (PCS) and mental health component score (MCS) of the SF-36. Also odds ratios of each parameter were calculated. Therefore, patients were classified into a group of poor and good responders for 6MWD (cut-off: Δ30m) and SF-36 (cut-offs: ΔPCS: 2.7; ΔMCS: 4.0). For group comparison, a 1:1-matching from an own retrospective COPD cohort (*n* = 708) was performed using FEV1 +/− 5%. Five LAM patients could not be assigned to an COPD patient. Therefore, a matching partner was assigned manually so that the mean values of FEV1% predicted did not differ significantly.

All statistical analyses were conducted using SPSS 23 (IBM, Inc., USA).

## Results

**Fig. 1:**
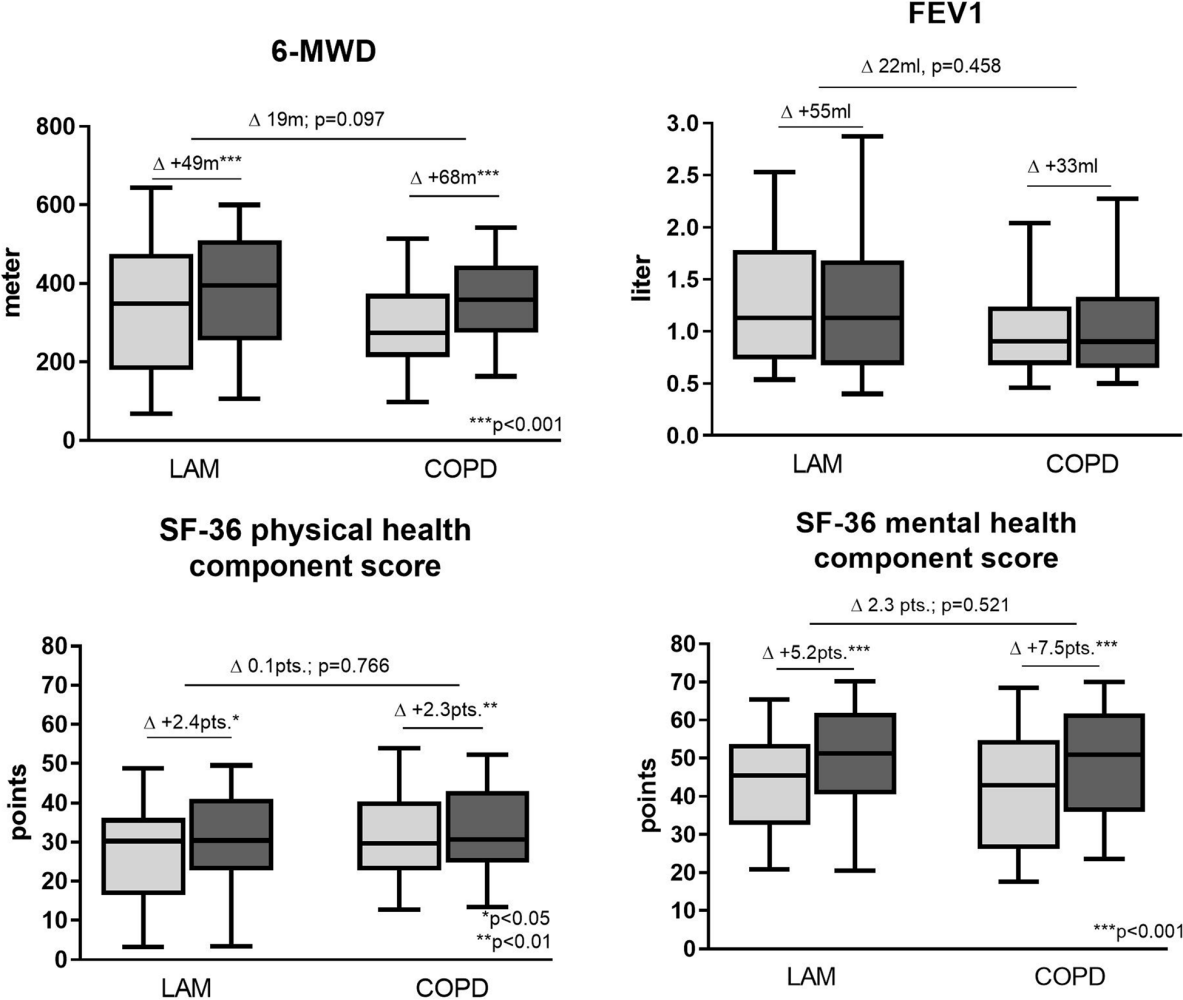
Outcome measures before (light grey boxplots) and after (dark grey boxplots) a 4-week pulmonary rehabilitation program (PR) in 58 LAM and 58 COPD patients for changes in 6-min walk distance (6MWD), forced expiratory volume in 1 s (FEV1) and quality of life (SF-36 questionnaire)

A total of *n* = 85 female patients with LAM performed PR during the observational period. However, *n* = 27 patients were excluded from the analysis due to following reasons: *n* = 21 (78%) performed repeated PR, *n* = 3 (11%) had missing data, and *n* = 3 (11%) were referred to another hospital due to acute clinical problems or worsening. The remaining 58 LAM patients showed a severely impaired lung function and exercise performance (Table 1). Eighteen patients (31%) received an mTOR inhibitor therapy with doses of 1.9 ± 0.5 mg. Following PR, 6MWD increased significantly by 49 ± 50 m (Fig. 1) exceeding the minimal important difference of 30 m [8]. Also quality of life (SF-36) improved significantly following PR (Fig. 1). These benefits were similar to the improvements in the COPD cohort. Results of the logistic regression analysis showed that none of the included variables (anthropometrics, lung function, mTOR inhibitor therapy, 6MWD or SF-36) was a significant predictor related to the improvements in 6MWD or quality of life. No exercise-related serious adverse events were recorded.

**Table 1:** Baseline characteristics of LAM patients and a COPD comparison cohort (data are presented as mean ± SD and [median])

	LAM	COPD	*p*
n	58	58	–
Female sex, n	58 (100%)	58 (100%)	–
Age, ys	48.2 ± 10.3 [48.4]	59.9 ± 11.6 [59.4]	< 0.001
BMI, kg/m^2^	22.9 ± 5.9 [21.6]	24.60 ± 7.19 [22.4]	0.071
Smoking status, never/former/current/unknown	35/17/2/4	7/45/6/0	< 0.001
FEV1, l	1.32 ± 0.74 [1.13]	1.00 ± 0.45 [0.91]	0.043
FEV1, %predicted	45.8 ± 24 [42.8]	45.4 ± 21.7 [41.5]	0.965
IVC, l	2.5 ± 1.0 [2.3]	1.9 ± 0.7 [1.9]	0.003
IVC, %predicted	72.1 ± 24.9 [74.4]	51.2 ± 15.9 [53.4]	0.001
DLCO, %pred.	40.7 ± 17.8 [37.2]	44.3 ± 17.1 [43.4]	0.481
PaO2, mmHg	65.7 ± 12.1 [63.9]	59.0 ± 10.4 [59.5]	< 0.001
PaCO2, mmHg	35.3 ± 5.2 [34.1]	41.0 ± 8.8 [38.4]	< 0.001
CRP, mg/l	3.2 ± 4.0 [2.0]	12.5 ± 28.0 [4.2]	< 0.001
Creatinine, mg/dl	0.76 ± 0.24 [0.74]	0.82 ± 0.23 [0.80]	0.028
Long-term oxygen therapy, n	36 (62%)	41 (71%)	0.326
Oxygen supplementation at rest, lpm	1.4 ± 1.5 [1]	1.2 ± 1.1 [1.5]	0.766
Oxygen supplementation during exercise, lpm	2.4 ± 1.9 [2]	1.8 ± 1.4 [2.0]	0.085
6MWD, m	338 ± 167 [330]	287 ± 121 [275]	0.097
6MWD, %predicted	47.7 ± 22.7 [48.3]	47.1 ± 20.2 [42.1]	0.850
SpO_2_ nadir during 6MWD	79.4 ± 6.8 [84]	84.9 ± 7.9 [87]	< 0.001
Listed for lung transplantation, n	28 (48%)	23 (40%)	0.350
SF-36 physical health component score	26.9 ± 12.8 [30.2]	30.8 ± 11.7 [29.6]	0.139
SF-36 mental health component score	44.0 ± 13.5 [45.4]	41.2 ± 16.3 [42.8]	0.359

Abbreviations: *BMI* Body mass index, *FEV1* Forced expiratory volume in 1 s, *IVC* Inspiratory vital capacity, *DLCO* Diffusion lung capacity for carbon monoxide, *PaO2* Partial pressure of oxygen, *CRP* C-reactive protein, *6MWD* 6-min walk distance

## Discussion

To the best of our knowledge this is the largest cohort of LAM patients, who were systemically analyzed before and after a comprehensive PR. To date, there is only one randomized, controlled trial available that investigated the effects of PR in 37 LAM patients [9]. In line with our results Araujo et al. found also significant improvements in exercise performance and quality of life. However, LAM patients that were included in that study had a much milder disease compared to patients in our study (FEV1: 72% and 6MWD: 517 m). Therefore, our study adds new evidence that PR is a beneficial treatment option even in patients with advanced LAM including patients listed for lung transplantation. Furthermore, we found that PR benefits were similar to the ones of a COPD cohort with similar disease severity. Until now, the best evidence for PR benefits is available for COPD patients [5]. In contrast, current LAM treatment guidelines either don’t mention PR as a treatment option [1] or state that PR may be offered to patients with LAM who are limited by dyspnoea (evidence level: expert opinion) [10]. The current ATS/ERS statement on PR lists LAM as a condition that may be appropriate for referral to PR. But this recommendation based only on extrapolating PR benefits from other chronic respiratory diseases and not on data [5].

PR had to be stopped in three patients (*n* = 1 pneumothorax, *n* = 1 acute worsening of dyspnea, and *n* = 1 pleural effusion) who needed to be transferred to an acute hospital. We interpret these events to be part of the natural course of the disease rather than to be related to any intervention during PR. In general, PR and in particular exercise training were regarded as feasible and safe in patients with advanced LAM.

Our study has some limitations. First, the COPD comparison group was significantly older, since not sufficient young COPD patients with severe airflow limitation were available. Second, data was collected using a retrospective study design over an observational period of two decades which is a rather long period given the fact that LAM is an orphan disease and medical treatment with mTOR inhibitors might have induced a bias during the last decade of our data collection. However, we could not identify any variability of PR effects over time.

## Conclusion

This study investigated the effects of PR in LAM. We found significant and clinically relevant improvements in exercise performance and quality of life following PR. Based on our systematic analyses of available data we recommend PR as a treatment option also for patients with LAM - a rare disease without many therapeutic options.

## Data Availability

The datasets used and/or analysed during the current study are available from the corresponding author on reasonable request.

## References

[1] McCormack FX, Gupta N, Finlay GR, Young LR, Taveira-DaSilva AM, Glasgow CG (2016). Official American thoracic society/Japanese respiratory society clinical practice guidelines: lymphangioleiomyomatosis diagnosis and management. Am J Respir Crit Care Med.

[2] Bahmer T, Watz H, Waschki B, Gramm M, Magnussen H, Rabe KF (2016). Reduced physical activity in lymphangioleiomyomatosis compared with COPD and healthy controls: disease-specific impact and clinical correlates. Thorax.

[3] Baldi BG, Albuquerque AL, Pimenta SP, Salge JM, Kairalla RA, Carvalho CR (2012). Exercise performance and dynamic hyperinflation in lymphangioleiomyomatosis. Am J Respir Crit Care Med.

[4] Johnson J, Johnson SR (2019). Cross-sectional study of reversible airway obstruction in LAM: better evidence is needed for bronchodilator and inhaled steroid use. Thorax.

[5] Spruit MA, Singh SJ, Garvey C, ZuWallack R, Nici L, Rochester C (2013). An official American thoracic society/European respiratory society statement: key concepts and advances in pulmonary rehabilitation. Am J Respir Crit Care Med.

[6] Gloeckl R, Marinov B, Pitta F (2013). Practical recommendations for exercise training in patients with COPD. Eur Respir Rev.

[7] Holland AE, Spruit MA, Troosters T, Puhan MA, Pepin V, Saey D (2014). An official European respiratory society/American thoracic society technical standard: field walking tests in chronic respiratory disease. Eur Respir J.

[8] Singh SJ, Puhan MA, Andrianopoulos V, Hernandes NA, Mitchell KE, Hill CJ (2014). An official systematic review of the European respiratory society/American thoracic society: measurement properties of field walking tests in chronic respiratory disease. Eur Respir J.

[9] Araujo MS, Baldi BG, Freitas CS, Albuquerque AL, Marques da Silva CC, Kairalla RA (2016). Pulmonary rehabilitation in lymphangioleiomyomatosis: a controlled clinical trial. Eur Respir J.

[10] Johnson SR, Cordier JF, Lazor R, Cottin V, Costabel U, Harari S (2010). European respiratory society guidelines for the diagnosis and management of lymphangioleiomyomatosis. Eur Respir J.

